# Prevalence and Radiographic Features of Head and Neck Soft Tissue Calcifications on Digital Panoramic Radiographs: A Retrospective Study

**DOI:** 10.7759/cureus.46025

**Published:** 2023-09-26

**Authors:** Aydan Acikgoz, Ozlem Akkemik

**Affiliations:** 1 Department of Dentomaxillofacial Radiology, Yeni Yuzyil University, Faculty of Dentistry, Istanbul, TUR; 2 Department of Dentomaxillofacial Radiology, Baris Medical Imaging Center, Izmir, TUR

**Keywords:** interpretation, soft tissue calcification, head and neck, maxillofacial region, panoramic radiography

## Abstract

Background

In this study, we aimed to determine the prevalence and radiographic features of incidental head and neck soft tissue calcifications (STCs) on panoramic imagesand assess their clinical significance.

Methodology

Following well-established training and calibration procedures, 9,553 digital panoramic radiographs (DPRs) taken between January 1, 2021, and January 31, 22, were retrospectively evaluated. Only obvious calcifications and clear differential diagnoses were considered. The presence, type, side (i.e., unilateral or bilateral), number (single or multiple), and the presence of different calcifications in the same individual were recorded. STCs were recorded according to age and gender. Data were analyzed using the chi-square test and Fisher’s exact test using SPSS version 18.0 (IBM Corp., Armonk, NY, USA).

Results

Overall, 35.8% of the DPRs studied showed the presence of STCs, including ossified stylohyoid complex (OSHC) (10.3%), thyroid cartilage (9.8%), tonsillolith (9.2%), atherosclerotic plaques (5.8%), calcified triticeous cartilage (CTC) (5.1%), sialolith (1.9%), as well as intra-articular (1.3%) and other calcifications (0.1-0.8%), i.e., calcified lymph node, antrolith, rhinolith, phlebolith, and osteoma cutis. STCs were found to be more prevalent in middle-aged patients and in females. A significant relationship was identified between the presence of carotid artery calcification and calcified superior horn of thyroid cartilage (CSHTC), as well as between the presence of CSHTC and CTC. Calcifications were detected either bilaterally (n = 2,003) or unilaterally (n = 2,388); however, OSHC mostly showed bilateral calcifications (8.5%).

Conclusions

Panoramic radiographs of dental patients reveal the frequent occurrence of STCs in the head and neck region with differing radiographic features. Certain calcifications show gender and age differences. Accurate detection of STCs may guide the identification of potential underlying diseases and help initiate referral to the relevant multidisciplinary teams.

## Introduction

Soft tissue calcifications (STCs) can have different appearances, locations, and causes. The irregular deposition of calcium in soft tissues is termed heterotopic calcification and can be divided into the following types: calcium deposition in damaged soft tissues as a result of an inflammatory response, trauma without mineral imbalance (dystrophic calcification), calcium deposition in normal tissues without mineral imbalance as a result of an idiopathic deposition (e.g., phleboliths), or calcium deposition in normal tissues due to mineral imbalance (metastatic calcification). Heterotopic calcification may develop in a wide variety of unrelated disorders and degenerative processes [1].

Incidental findings such as calcifications may occur in certain soft tissue diseases, but the lesions may or may not correspond to pathological findings. Some STCs do not require any intervention or long-term surveillance, while others can be life-threatening with the underlying cause requiring treatment. Imaging methods such as MRI, CT, ultrasound, and even histological examination may be insufficient for the diagnosis of calcifications in some soft tissue-associated diseases [2]. Recently, the American College of Radiology (ACR) revised the criteria for imaging soft tissue masses. Accordingly, asymptomatic STCs were deemed to not justify the use of advanced three-dimensional imaging methods that use high-dose radiation [3]. Therefore, it is imperative to accurately identify calcifications and determine if treatment or further investigation is needed.

The importance of radiographs in the complete characterization of musculoskeletal pathologies has been emphasized. For example, MRI shows various signal intensities on T1 or T2-weighted images in conventional spin echoes and can often miss calcifications. Therefore, for the full characterization of musculoskeletal pathologies, it is important to compare MRI with conventional radiographs, which can identify calcifications more accurately [1,4]. In the event of an incidental finding in an asymptomatic patient, the clinician is expected to examine the radiographic features of the calcifications with knowledge of the radiographic anatomy. In this manner, further follow-up of the patient or referral to the relevant specialties can be provided.

Panoramic radiography (PR), in which the neck region is also monitored, is commonly used when clinical imaging of the entire maxillofacial region (MFR) is required. Dental radiology studies have extensively reported on the calcifications in the MFR or head and neck region (HNR) and their radiological properties [5]. However, to our knowledge, such studies are not exhaustive and have not been conducted using a large number of samples. Population imaging studies play an important role in radiology by enhancing the robustness of the data. Furthermore, prevalence studies provide data on the most frequently observed abnormalities, encouraging the physician to investigate further.

According to the guidelines provided by ACR, the lack of comparative radiographs may lead to misinterpretation, additional/unnecessary imaging, or unnecessary treatments [3]. Radiography is generally the first examination technique when imaging is clinically indicated. Considering this, we systematically examined and compared STCs in full-sized PRs in a large population of patients. If present, we aimed to identify and define the type of STC based on their radiographic features (location, sidedness, number), demographic characteristics (age-gender distribution), the existence of different calcifications in the same individual, and assess their clinical significance.

## Materials and methods

The protocol of this retrospective study was approved by the Yeni Yuzyil University School of Medicine Non-interventional Clinical Research Ethics Committee (approval number: 2022/04-838) and was conducted in accordance with the Declaration of Helsinki 1975, revised in 2013. All patients provided signed informed consent.

Study population

Digital panoramic radiographs (DPRs) recorded between January 2021 and January 2022 (January 1, 2021, to January 31, 2022) were evaluated in this study. None of the patients were exposed to any extra radiation because of the study. DPRs, which were requested because they were deemed necessary for dental treatment, were retrieved from the archives of the Maxillofacial Radiology Department and evaluated. Demographic characteristics of the patients (age, gender) were recorded. The study population was divided into three groups according to their age range: Group 1 (young, 18- 30 years), Group 2 (middle-aged, 31-50 years), and Group 3 (elderly, >51 years). The inclusion criteria were patients aged 18 years or older with no history of surgery, trauma, or pathology in the MFR, and the availability of radiographs showing the HNR up to the fourth cervical vertebra. The exclusion criteria were images of poor quality, with the presence of distortions or artifacts.

Image acquisition

All DPRs were obtained using a Vatech Pax -i 2D X-ray digital panoramic unit (Vatech, Seoul, Korea) under a standard exposure factor (50-90 KVp/4-10 mA), as recommended by the manufacturer. All images were analyzed using the image-processing program EzDent-i 2D viewer software (Tip Plus Hospital Automation Software), allowing the adjustment of contrast and the use of magnification tools. Panoramic images were evaluated using a standard computer monitor (22-inch LCD with a resolution of 1,920 × 1,080 pixels) in ambient light ranging from 300 to 500 lux. The monitor’s brightness setting was automatically set to a low fixed value, which helped preserve the image detail and contrast. This setting provided a standard brightness level even in low ambient light conditions. Measurements were performed with the help of an editing tool available in the Turcasoft software [6].

Panoramic image analysis

PR images of patients with calcifications were investigated for the type of calcification and sidedness (bilateral or unilateral). Single or multiple occurrences of the same type of calcification were recorded. If diverse types of calcifications were seen in the same individual, each calcification was classified individually. If present, the types of calcifications were noted as a single type, two types, or multiple types. STCs in the HNR that were examined in this study are shown in Table 1 [7-19]. The length of the stylohyoid process (SP) was measured as the distance from its base (tympanic plate) in the temporal bone to its tip. SPs longer than 30 mm were considered to be ossified stylohyoid complex (OSHC). Classification according to the patterns of OSHC was not attempted in this study. Radiopaque patterns encountered on the DPRs that did not correspond to any category were marked as undefined. Only obvious calcifications and clear differential diagnoses were considered. The findings were correlated with age and sex.

**Table 1:** Definition of soft tissue calcifications, radiographic features, and location in radiographs.

Calcification		Definition	Location	Radiographic features
Stylohyoid Chain[7,8]		Elongated stylohyoid process and all types of ossified stylohyoid ligament together are referred to as “ossified stylohyoid complex”	The bone and ligament complex that extends from the base of the temporal bone styloid process to the lesser horn of the hyoid bone	Variations of the stylohyoid complex: - Various lengths of stylohyoid process - Various degrees of ossification of stylohyoid ligament - Various fusions of parts of the stylohyoid complex
Laryngeal cartilages [9]	Superior horn of thyroid cartilage	The largest cartilage of the larynx	Below the hyoid bone	Usually appears medial to C4 at the lower edge of the panoramic radiography
	Triticeous cartilage	A rare laryngeal skeletal formation with an incidence rate of 12–65%	Within the thyrohyoid ligament between the greater horn of the hyoid bone and the superior horn of thyroid cartilage	When calcified, appears as mostly oval with smooth edges, with well-defined cortical margins at the level of the third and fourth cervical vertebrae
Carotid artery calcification [10]		Atherosclerotic plaques that are detected in the main bifurcations of the carotid artery	Adjacent to the cervical vertebrae at the level of the C3-C4 intervertebral junction	A radiopaque vertical line or nodular radiopaque mass
Tonsillolith [11,12]		Calcified tonsil within enlarged tonsillar crypts	Due to the angulation of panoramic X-ray projection, tonsillolith can observed in different regions including along the inferior two-thirds of the mandibular ramus; overlap either with the anterior or posterior of the ramus; posteroinferior to the angle of the mandible	Usually appear as either multiple clusters of irregular radiopacities or as a solitary round radiopaque structure
Sialolithiasis [13] (salivary stones)		Calcified mass within a salivary gland	- Submandibular stones: above or below the mandibular body, as well as mesial to the mandibular angle - Parotid stones: in the upper third of the ramus or located behind the lower third of the ramus or behind the angle of the mandible	Almost always unilateral, solitary, or multiple as diffuse, homogeneous, uniformly calcified radiopacities with regular contours. If large, showing multiple layers of calcification
Loose intra-articular bodies in the temporomandibular joint [14,15]		Free-floating calcified masses in the joint space; develop by certain alterations of the temporomandibular joint including osteoarthritis, osteochondritis dissecans, synovial chondromatosis, osteochondroma, chondrocalcinosis, intracapsular fractures	In the temporomandibular joint space	Radiopaque structure/s in the temporomandibular joint space/s
Peripheral lymph nodes [16]		A complex component of the immune system including cervical, submandibular, submental, and preauricular lymph nodes	Nearly half of all peripheral lymph nodes are located deep in the subcutaneous tissue in the head and neck	Multiple, irregular opacities (cauliflower appearance); observed most often in the submandibular region, posterior to the inferior third of the ramus or inferior to the angle of the mandible
Phlebolith [17]		Calcification of intravascular thrombus	The parotid gland is the major site (85%) of vascular malformations that affect the salivary glands	Resembles sialolith; multiple random circular radiopacities with a laminated morphology and a radiopaque or radiolucent center
Osteoma cutis[18]		Bone formation within the skin	Usually appears on the face	Consistent with bone density, doughnut or snowflake-like or washer-shaped
Mönckeberg arteriosclerosis [19]		Arteriosclerosis characterized by the formation of calcium deposits, particularly in the media layer of peripheral arteries	Unlike carotid artery calcification, mönckeberg arteriosclerosis’ are not focal plaques; rather, they extend along the greater length of the arteries	“Pipe-stemming, or railroad tracks” appearance as a parallel pair of thin, radiopaque lines
Rhinolith/Antrolith [6]		Calcified bodies within the antral/nasal cavity. Endogenous (sequestra, blood, tooth etc.) or exogenous (food, root fragments, etc.)	In the maxillary sinus (antrolith) or in the nasal cavity (rhinolith), typically between the nasal septum and inferior turbinate	Homogeneous or heterogeneous radiopacities depending on the nature of the nidus

Training and calibration procedures

The radiographs were examined by seven different observers. Among them was an Oral and Maxillofacial Radiologist with over 30 years of clinical experience in the MFR (reference examiner: consultant, AA), along with six newly graduated dentists (observers). Instruction criteria for the radiographic assessment of the calcifications were defined by the consultant. Before the calibration process, the observers were trained at two-week intervals by the consultant using representative images based on previous studies and the relevant literature. After the training, a pilot study was conducted by the observers on the images of 74 cases with different types of calcifications twice, two weeks apart, to ensure intra and interexaminer reliability. Each observer, using separate computers but with the same conditions, was asked to evaluate the types of STCs on the images. No limitation was imposed on the time required to examine the images. The overall interobserver agreement ranged from 57.6% to 83.8% in the first assessment, and from 81.8% to 89.9% in the second assessment, showing almost perfect agreement on the diagnosis of STCs (Table 2).

**Table 2:** Reliability values of the observers on panoramic film scan scores calculated by kappa statistics before and after training. Cohen’s kappa was interpreted as follows: 0.01-0.20, none to slight; 0.21-0.40, fair; 0.41-0.60, moderate; 0.61-0.80, substantial; and 0.81-1.00, almost perfect agreement.

Observers	First reading					Second reading				
	Κ	SE	Overall agreement (%)	P-value	IC-95%	κ	SE	Overall agreement (%)	P-value	IC-95%
#1	0.284	0.101	78.8	0.000	0.086-0.482	0.627	0.110	81.8	0.000	0.311-0.743
#2	0.402	0.110	83.8	0.000	0.187-0.617	0.679	0.119	84.8	0.000	0.246-0.713
#3	0.208	0.083	71.7	0.000	0.046-0.369	0.721	0.125	86.9	0.000	0.376-0.866
#4	0.113	0.055	57.6	0.036	0.005-0.221	0.711	0.115	85.9	0.000	0.416-0.866
#5	0.323	0.097	78.8	0.000	0.134-0.513	0.721	0.125	86.9	0.000	0.376-0.866
#6	0.298	0.091	76.8	0.000	0.119-0.477	0.837	0.123	89.9	0.000	0.496-0.978

Subsequent to the training and calibration process, a cumulative study on 9,553 patients was conducted. The DPRs recorded between January 2021 and June 2021 were distributed equally among the observers. The DPRs recorded between June 2021 and January 2022 were analyzed by the consultant. Each observer, including the reference examiner, re-examined the images with an interval of at least three weeks between their assessments. In case of doubt, the reference examiner was consulted for the final decision. If still in doubt, the diagnostic workup included consultation with another senior maxillofacial radiologist (OA).

Statistical analysis

The data were tabulated in an Excel spreadsheet and analyzed using the SPSS software version 25.0 (IBM Corp., Armonk, NY USA). The distribution of gender and age as well as the relationship between gender and age and clinical findings were analyzed using the chi-square test or Fisher’s exact test. Kappa statistic was used to determine consistency and interobserver reliability between the consultant and the observers. Statistical significance was evaluated as p-values <0.05.

## Results

DPRs from a total of 9,553 patients (41.1% males, 58.9% females, age range of 18-93 years) without any clinical data were examined retrospectively. Overall, 45.1% of the patients were between the ages of 31 and 50 years; 58.9% of these were female.

Calcifications were detected in 35.8% of the patients. Among these patients, 38.0% were male and 62.0% were female. The mean age of the patients with calcification was 44.34 ± 13.69 years and without calcification was 39.24 ± 14.34 years (Table 3).

**Table 3:** Demographic characteristics of the study group and distribution of calcifications by gender, age, and presence of different types of calcifications in the same person. OSHC: ossified stylohyoid chain; CSHTC: calcified superior horn of thyroid cartilage; CAC: carotid artery calcification; CTC: calcified triticeous cartilage; CLN: calcified lymph node; LB loose bodies in the temporomandibular joint

		Male, n (%)	Female, n (%)	Age Group I (18–30)	Age Group II (31–50)	Age Group III (>51)
Demographic characteristics, Total n = 9,553		3,926 (41.1%)	5,627 (58.9%)	2,658 (27.88%)	4,308 (45.1%)	2,587 (27.1%)
Patients having calcifications, Total n = 3,418 (35.8%)		1,300 (38.0%)	2,118 (62.0%)	618 (18.1%)	1,662 (48.6%)	138 (33.3%)
STC types in the same person	Single type, n (%) = 2,575 (75.3%)	1,028 (39.9)	1,547 (60)	518 (20.1%)	1,229 (47.7%)	828 (32.2%)
	Two types, n (%) = 733 (21.5%)	241 (32.9)	492 (67.1)	93 (12.7%)	378 (51.6%)	262 (35.7%)
	Multiple types, n (%) = 110 (3.2%)	31 (28.2%)	79 (71.8%)	7 (6.4%)	55 (50.0%)	48 (43.6%)
OSHC		392 (39.7)	596 (60.3)	229 (23.2%)	457 (46.3%)	302 (30.5%)
CSHTC		238 (25.5)	696 (74.5)	102 (10.9%)	521 (55.8%)	311 (33.3%)
Tonsil stone		463 (52.9)	413 (47.1)	152 (17.4%)	384 (43.8%)	340 (38.8%)
CAC		143 (25.9)	410 (74.1)	86 (15.6%)	265 (47.9%)	202 (36.5%)
CTC		139 (28.5%)	349 (71.5%)	45 (9.2%)	277 (56.8%)	166 (34.0%)
Sialolith		73 (41.0%)	105 (59.0%)	41 (23.0%)	89 (50.0%)	48 (27.0%)
LB in TMJ		45 (37.5%)	75 (62.5%)	19 (15.8%)	52 (43.3%)	49 (40.9%)
CLN		38 (50.0%)	38 (50.0%)	9 (11.8%)	32 (42.1%)	35 (46.1%)
Antrolith		389 (48.1%)	41 (51.9%)	22 (27.8%)	40 (50.65%)	17 (21.6%)
Osteoma cutis		1	9	3 (30.0%)	3 (30.0%)	4 (40.0%)
Phlebolih		0	1	-	-	1
Rhinolith		6 (46.2%)	7 (53.8%)	4 (30.8%)	4 (30.8%)	5 (38.4%)
Mönckeberg arteriosclerosis		2	-	-	1	1
Undefined		22 (43.1%)	29 (56.9%)	14 (27.5%)	20 (39.2%)	17 (33.3%)

**Figure 1:**
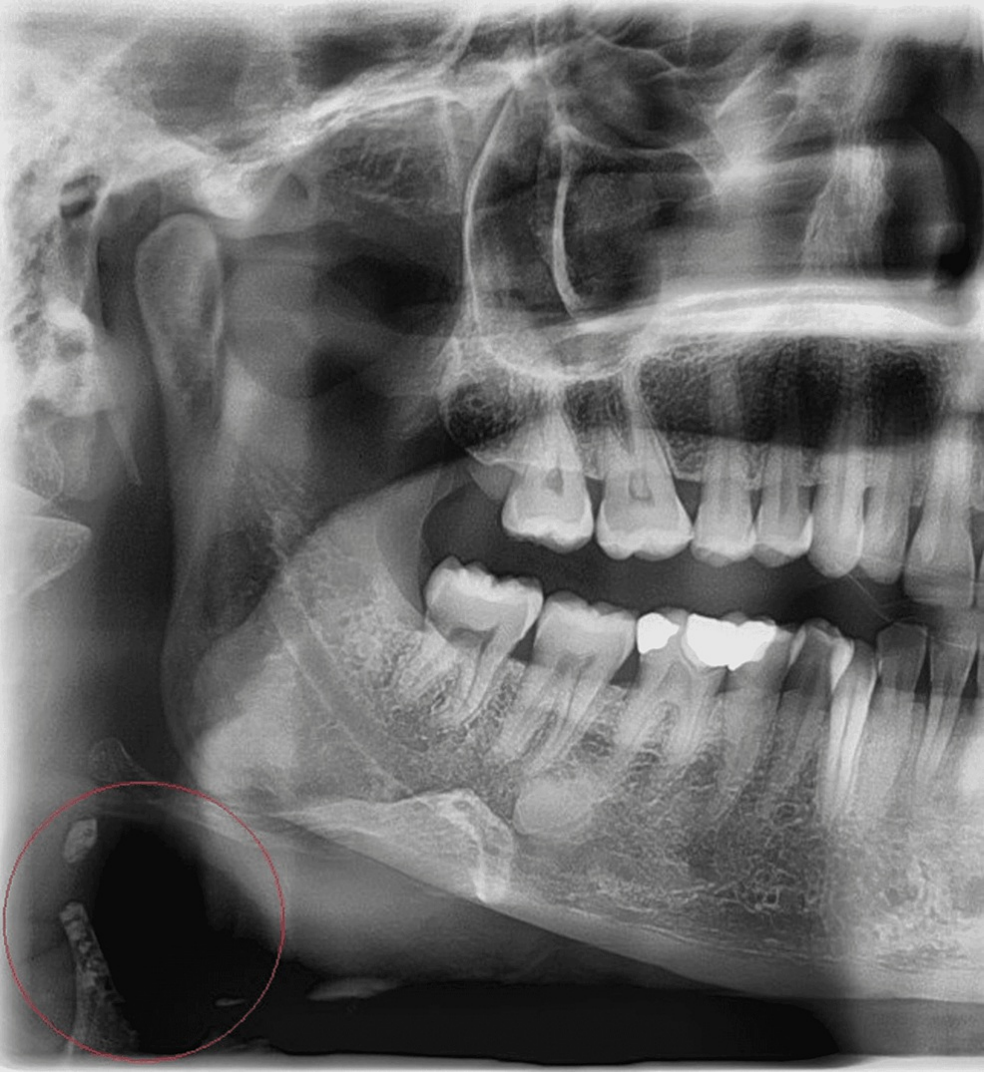
Calcified triticeous cartilage and calcified superior horn of the thyroid cartilage at the level of the third and the fourth cervical vertebrae in a 45-year-old woman.

**Figure 2:**
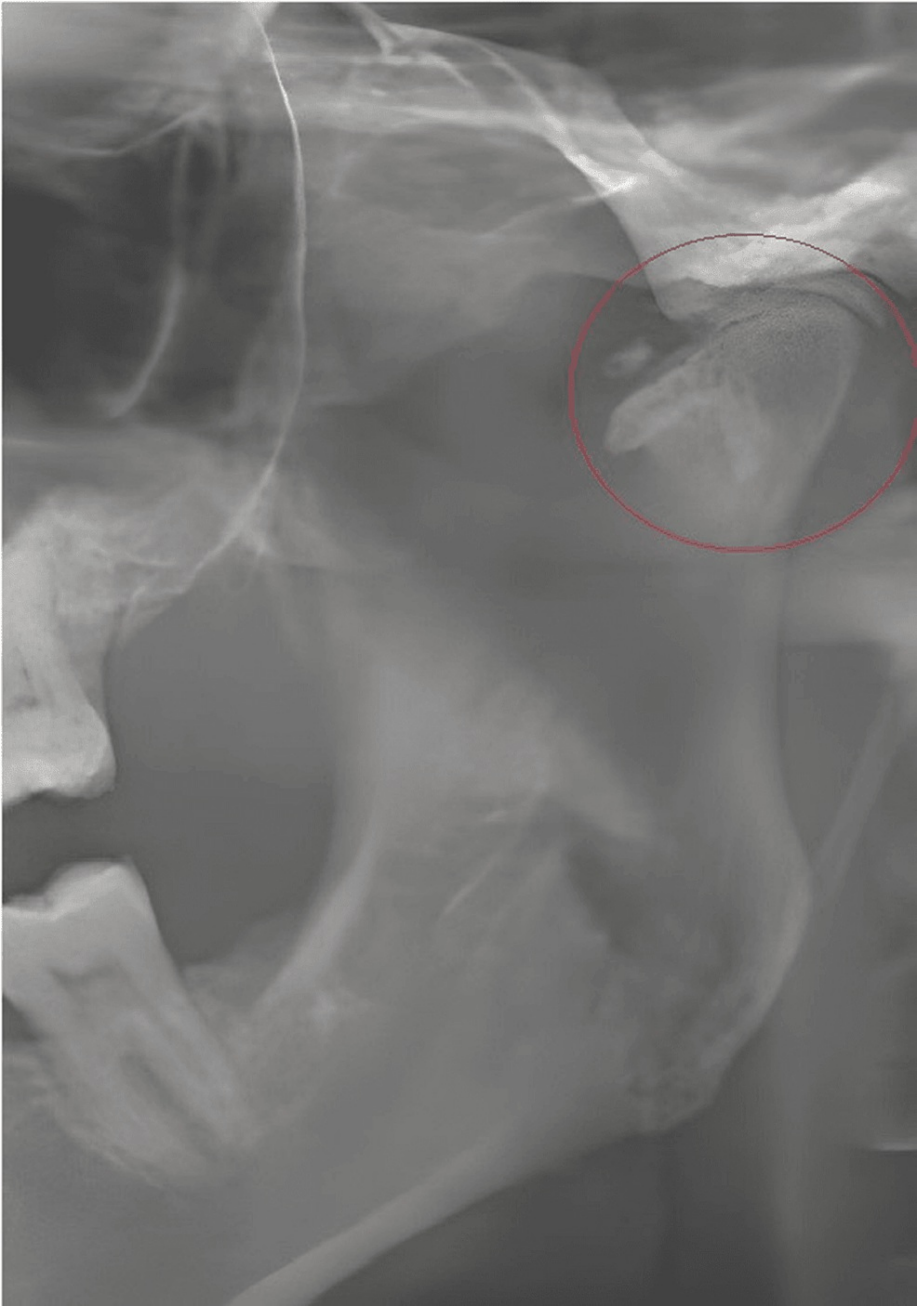
Panoramic radiography shows degenerative condyle changes and discrete radiopaque bodies in the left joint space of a 38-year-old woman.

The most prevalent STC in this study was OSHC (10.3%), followed by the calcified superior horn of thyroid cartilage (CSHTC) (9.8%), tonsillolith (9.2%), carotid artery calcification (CAC) (5.8%), calcified triticeous cartilage (CTC) (5.1%) (Figure 1), sialolith (1.9%), and temporomandibular (TMJ) calcifications (1.3%) (Figure 2). Other rare calcifications ranged from 0.1% to 0.8%, while 0.5% of the cases comprised undefined calcifications (Table 3).

**Figure 3:**
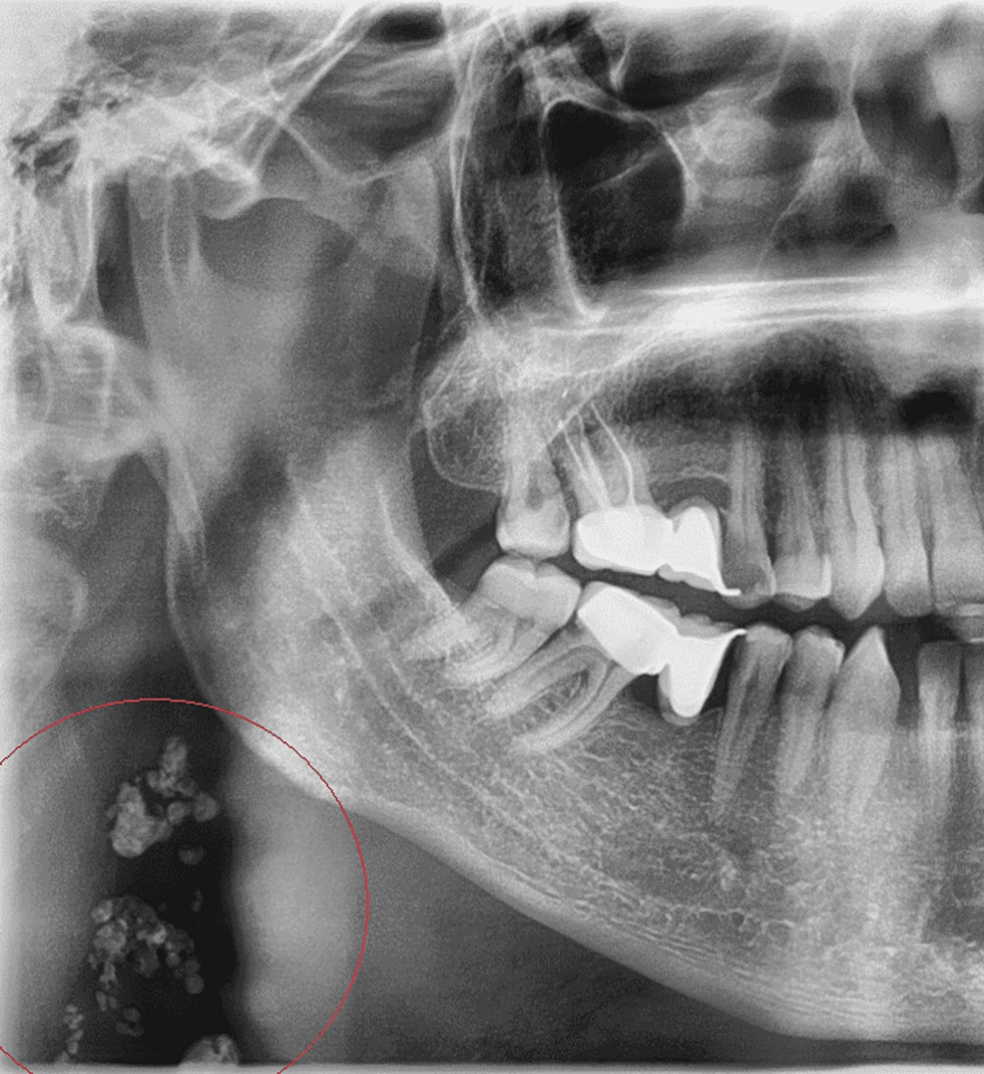
Panoramic radiography demonstrates multiple radiopaque masses consistent with calcified cervical lymph nodes under the right angulus mandible in a 44-year-old man.

A significant relationship was identified between the presence of specific calcifications, gender, and age of the patients. Tonsillolith was found more frequently in males, whereas CSHTC, CTC, and CAC were observed predominantly in females (Table 2). However, there was no statistically significant relationship between gender and the presence of STCs such as OSHC, sialolith, loose bodies (LBs) in TMJ, calcified lymph node (CLN), antrolith, osteoma cutis, phlebolith, and rhinolith (p > 0.05). The majority of cases diagnosed with OSHC, CSHTC, CTC, tonsillolith, CAC, LBs in TMJ, and CLN (Figure 3) were in the middle-aged group (Table 3).

**Figure 4:**
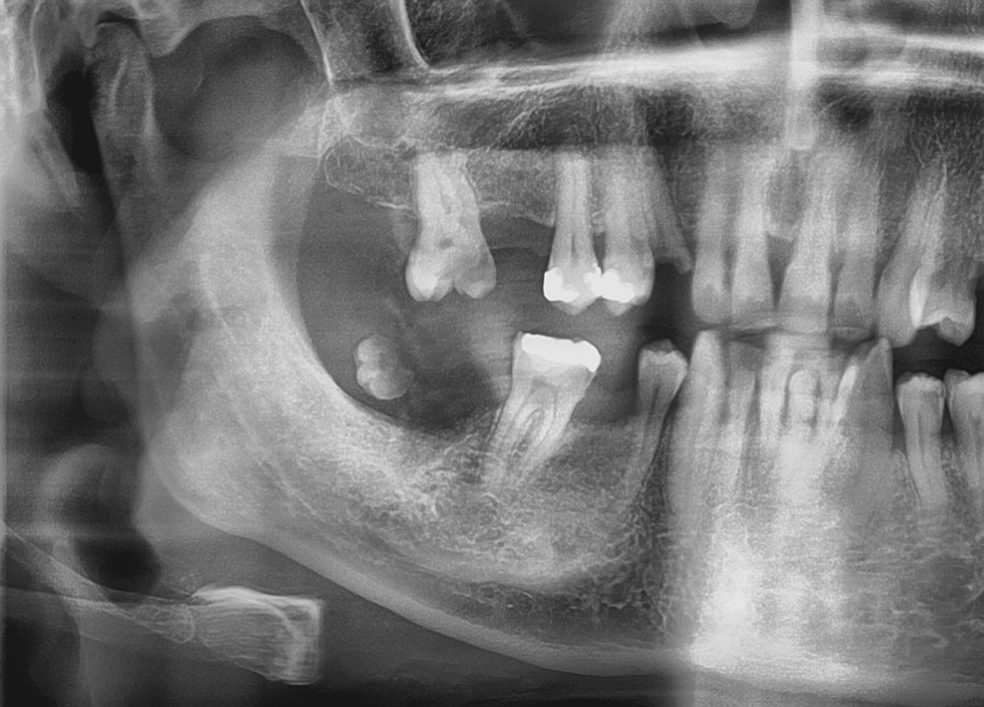
A radiopaque solitary nodule consistent with a large osteoma cutis on the cheek of a 40-year-old woman. Panoramic radiography shows a radiolucent center compatible with normal fatty bone marrow, but the normal trabecular structure is not observed. The periphery appears more radiopaque than the inferior.

**Figure 5:**
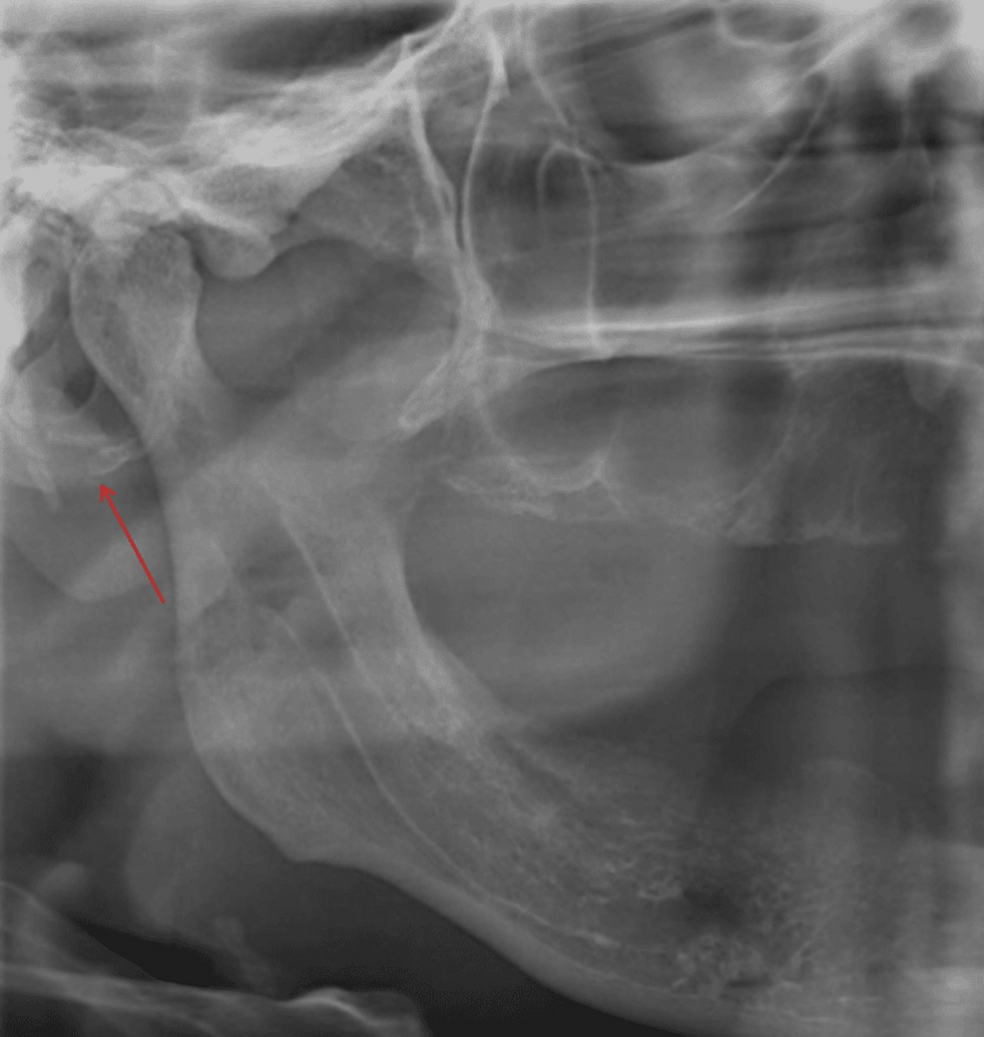
Panoramic radiograph shows a calcified maxillary artery at the level of the neck of the mandible in a 74-year-old man.

Statistical analyses showed a significant correlation in the presence of CAC and CSHTC. CAC was observed in 8.8% of patients with CSHTC (9.8% incidence rate). Furthermore, there was a significant relationship between the presence of CSHTC and CTC. Calcifications were detected either bilaterally (n = 2,003) or unilaterally (n = 2,388); however, OSHC was mostly observed to be calcified bilaterally (8.5%) (Table 4). Other STCs in the MFR such as CLN, antrolith, osteoma cutis (Figure 4), phleboliths, rhinolith, and Mönckeberg arteriosclerosis (MA) (Figure 5) were observed in the current sample, but their occurrence did not reach statistical significance (Table 3).

**Table 4:** Distribution and lateral pattern of soft tissue calcifications observed on panoramic radiographs. OSHC: ossified stylohyoid chain; CSHTC: calcified superior horn of thyroid cartilage; CAC: carotid artery calcification; CTC: calcified triticeous cartilage; CLN: calcified lymph node; MA: Mönckeberg arteriosclerosis; LB: loose bodies in the temporomandibular joint

Type of calcification	Total, n (%)	Right, n (%)	Left, n (%)	Bilateral, n (%)
OSHC	988 (10.3%)	114 (1.2%)	61 (0.6%)	813 (8.5%)
CSHTC	934 (9.8%)	372 (3.9%)	156 (1.6%)	413 (4.3%)
Tonsillolith	876 (9.2%)	274 (2.9%)	257 (2.7%)	354 (3.7%)
CAC	533 (5.8%)	244 (2.6%)	157 (1.6%)	154 (1.6%)
CTC	488 (5.1%)	204 (2.1%)	94 (1.0%)	193 (2.0%)
Sialolith	178 (1.9%)	87 (0.9%)	82 (0.9%)	9 (0.1%)
LB in TMJ	20 (1.3%)	43 (0.5%)	28 (0.3%)	49 (0.5%)
CLN	76 (0.8%)	31 (0.3%)	36 (0.4%)	9 (0.1%)
Antrolith	79 (0.8%)	40 (0.4%)	36 (0.4%)	3
Osteoma cutis	10 (0.1%)	7	2	1
Phlebolith	1 (0.1%)	-	1	-
Rhinolith	13 (0.1%)	7 (0.1%)	6 (0.1%)	-
MA	2 (0.1%)	1	1	-
Undefined	51 (0.5%)	28 (0.3%)	18 (0.2%)	5

## Discussion

According to the available literature, the prevalence of STCs in the MFR is in the range of 1.5% to 5.7% on PR images and between 16.4% and 43% on cone beam computed tomography (CBCT) images (Table 5) [20-29]). In this study, STCs were incidentally observed in 3,418 (35.8%) of 9,553 patients evaluated retrospectively; this rate was close to the value reported by Yalçın and Ararat [26].

**Table 5:** Prevalence rates of soft tissue calcifications identified by cone beam computed tomography and in the full size of panoramic radiography in reported studies. DPR: digital panoramic radiograph; STC: soft tissue calcification; OSHC: ossified stylohyoid complex; MAWC: mean age with calcification; MAWOC: mean age without calcification; y/o: years old; MX-FOV: maxilla; MD-FOV: mandible; MM-FOV: maxilla and mandible; Excl: except for; PWC: patients with calcification

Reference (year), total sample, n	Imaging modality + FOV	Age of total sample and MAWOC	Prevalence of calcification and MAWC	Most common type of calcification in PWC	Gender-age Comparison in PWC
Current study (2022), n = 9,533	Full size of DPR	18–93, MAWOC: 39.24± y/o	35.8% (n = 3418), AWC: 44.34±	OSHC (10.3%)	Females related to age and gender
Maia et al.(2021) [21], n = 1,176	Full size of DPR	>60 years, MAWOC: 67.47 y/o	43%, AWC: No report	CSHTC (23.6%)	Females
Akgunlu (2019) [22], n = 4,263	Full size of DPR	6–89 years, MAWOC: 27.44±	6.4% (n: 270), AWC: 40.37± y/o	Tonsillolith (2.5%)	No significant difference between genders; related to increasing age
Ribeiro et al.(2018) [23], n = 2,375	Full size of DPR	3–90 y/o, MAWOC: No report	19.7% (n: 420), AWC: No report	OSHC (13.1%)	Not gender related; related with increasing age
Bayramov et al. (2022) [24], n = 1,566	Maxillofacial CBCT	18–80 y/o, MAWOC: No report	24.9 % (n: 390), AWC: 47.3±	Tonsillolith (53.6%)	Different rates across age/gender groups depending on STC type
Elhadidy et al.(2021) [25], n = 417	Large FOV CBCT	10–80 y/o, MAWOC: No report	22.54% (n: 94), MAWC: 42.07±	OSHC (16.55%)	Higher in males; different rates across age groups depending on STC type
Yalcin and Ararat (2020) [26], n = 1,557	16 × 5, 16 × 9, 16 × 16 cm	11–84 y/o, MAWOC: 35.25±	33.4% (n: 520), MAWC: 44.22± y/o	Tonsillolith (18.8%)	Predominant in males, significantly higher in older patients
Missias et al. (2018), [27] n = 1,000	MX-FOV 57.2% MD-FOV 60.6% MM-FOV 76.8%	<30–≥ 60 y/o, AWOC: No report	62.6% (n: 626), MAWC: No report	Tonsillolith OSHC	No significant association with age or gender
Patil et al. (2017) [28], n = 624	Maxillofacial CBCT	Age range: MAWOC: 43.74± y/o	25.48% (n: 159), MAWC: 48.64± y/o	Arteriosclerosis (45.91%)	Predominant in females and advanced age
Wells and Adam (2011) [29], n = 308	MM-FOV MD-FOV Full 13.2 c m	7–86 years, MAWOC: 47.5± y/o	34.75% (n: 107), MAWC: 59.5	CTC (32.3%)	Predominance in females; calcifications found in older patients

The present study showed that the most common calcifications in the HNR were OSHC (10.3%), CSHTC (9.8%), tonsillolith (9.2%), CAC (5.8%), and CTC (5.1%). The prevalence rate of OSHC was lower than that reported in previous studies [7,24,25] and higher than the prevalence rate reported by Yalcin and Ararat [26] using CBCT images. CSHTC was found to be less frequent compared to other previously published PR or CBCT studies [21,29], whereas the incidence rate of tonsillolith was within the range reported in previous PR studies [21-23]. The prevalence rate of CAC in the present study was close to that reported by Ribeiro et al. [23]. The calcification rate of TC was similar to the CT findings of Alqahtani et al. [9], followed by sialolith (1.9%), and LB in TMJ (1.3%). Antrolith, CLN, Osteoma cutis, phlebolith, rhinolith, and MA were among the rarely observed calcifications.

Various studies suggest the presence of a gender and age bias in the development of calcifications [7-20]. In agreement with previous CBCT and PR studies [24,25,30], we found STCs to be related to both age and gender; however, our data was in contrast to the CBCT results of Misias et al. [27]. Consistent with several studies [21,24,28,29], but unlike others [25,26], the current work found that calcifications were more common in female patients. We found that some structures such as SHTC, TC, and CAC were more likely to be calcified in females. Age also was one of the major factors related to maxillofacial calcifications, which has been indicated in several other studies [21-23,26,28,29]. The present study found that calcifications of structures such as SHC, SHTC, TC, tonsils, lymph nodes, and LB in TMJ were detected more frequently in middle-aged patients.

The pattern of distribution of calcifications in the current study was also in accordance with the distribution described in the literature [24-26]. A unilateral pattern was observed more commonly than bilateral in this study including a unilateral predominance in the calcification of structures such as SHTC, tonsils, TC, and carotid artery.

Reports on the occurrence of diverse types of calcifications in the same individual are rare. In the current study, the incidence of single, double, and multiple calcifications in the same individual was 75.3%, 21.5%, and 3.2%, respectively. Thus, the formation of a single type of calcification was more common than the formation of multiple types of calcifications in the same patient, a finding that was corroborated by Elhadidy et al. [25] and Yalcin and Ararat [26].

Soft tissue calcifications of clinical importance

The present study found that some STCs that are (CAC (5.8%)) or might be (stylohyoid ligament calcification (10.3%), tonsillolith (9.2%), TMJ calcifications (1.3%)) of clinical importance. The common carotid artery lies in the focal trough of the PR. Previous studies have reported a 2%-12.5% prevalence rate of CACs on PRs in the dental patient population [10, 21, 22]. CAC may be useful in identifying asymptomatic at-risk individuals, who may then proceed with further investigations [10].

Some previous studies have excluded OSHC from the search criteria of calcifications detected in PR [21]. However, radiographic findings of the SH complex have been found to be relatively similar to CAC and LNC. A few studies have supported the association between osteoporosis, elongated SHL, and vascular calcification [8]. Additionally, a more recent study using CBCT and MRI showed that stylohyoid syndrome may be misdiagnosed as TMJ disorder [30]. We believe that these aforementioned studies underline the importance of examining the OSHC when evaluating calcifications on PR images. Although tonsilloliths are usually asymptomatic, tonsil stones should be kept in mind in patients with oropharyngeal discomfort [20].

Numerous conditions of the joint are associated with TMJ calcifications, including TMJ osteoarthritis, desiccant osteochondritis, synovial chondromatosis, chondrocalcinosis, rheumatoid arthritis, and intracapsular fractures [14]. Therefore, referrals for the evaluation of underlying causes upon incidental observation of such calcifications may benefit the diagnosis and prognosis of affected patients.

Phleboliths may appear separately or may be accompanied by hemangioma, which may require monitoring in subsequent studies. Pathogenesis may be related to an injury to the vessel wall or stagnation of blood flow due to trauma or vascular malformations [17].

MA mostly occurs in the lower limbs and visceral arteries; however, calcifications in the HNR have also been reported. MA is mostly seen in older male patients and is associated with uncontrolled diabetes and chronic renal failure [19]. Although observed with a low incidence rate in this study, phleboliths and MA are conditions that need to be monitored.

Asymptomatic small sialoliths are usually benign and do not require treatment while large stones require further investigation. In this study, the prevalence rate of CLN was found to be lower than in previously reported studies [24]. Lymph node calcifications are generally benign; nonetheless, it is recommended to further examine extensively calcified lymph nodes as they may be active [29].

Ossified stylohyoid chain

The bilateral pattern of OSHC is a consistent finding in many studies. Aging has also been reported as a consistent factor for OSHC by other researchers, supporting the results of the current study; however, there are controversial reports on gender bias [24,25]. This study showed no association between OSHC and gender, similar to other reported studies [23,25].

Calcified superior horn of thyroid cartilage

CSHTC is a natural process and increases with age, suggesting a progressive nature of this calcification [21,25,29]. It also corroborates with the data from the current study showing that middle-aged patients were significantly more affected by CSHTC. Consistent with the findings of previous studies, CSHTC was found to undergo calcification to a greater extent in the female population. Furthermore, we detected CSHTCs either unilaterally (5.5%) or bilaterally (4.3%), while Elhadidy et al. and Wells reported a bilateral occurrence of CSHTC [25,29].

No treatment is generally required for calcified laryngeal cartilage. However, it is still important to know their incidence, shape, and location because these structures can often be mistaken for pathosis in this region.

Calcified triticeous cartilage

Unlike the data reported by Alqahtani et al. [9], but similar to other studies [21,25,29], we found an association between gender, age, and incidence of CTC. Previous 3D studies have demonstrated a predominance of bilateral CTC formation, contradicting the results of the current study [9,25,28]. A significant correlation between the formation of CTC and CSHTC has been reported, supporting our data, and suggesting that both structures undergo similar changes [21,29].

Carotid artery calcification

We found CAC to be more common in females and older patients, which is supported by some [21,23,25] but refuted by other studies [4]. We found frequent unilateral occurrence of arterial calcifications consistent with the findings of previous studies [25] and contrary to some others [24,29]. In this study, older patients with CAC showed a significant association with CSHTC (p < 0.05), supporting a function of age in the incidence of both conditions.

Phleboliths

The true incidence of phleboliths in the MFR is unknown due to its rarity and the infrequent occurrence of symptoms [24,27,28]. Among the 9,533 DPRs examined in the current study, we detected multiple phleboliths in the oropharyngeal region in an older patient.

Mönckeberg arteriosclerosis

CAC and MA can be differentiated based on the location and appearance of the calcifications. In the current study, maxillary artery calcification (branched from the external carotid artery) was observed behind the neck of the mandible in two male patients.

Tonsillolith

Tonsil stones may occur bilaterally while unilateral lesions may generate a ghost image on the contralateral [11,12,20]. Corroborating the data from several previous studies, we observed that the formation of unilateral tonsillolith was more common than bilateral [11,12,28,30]. Furthermore, similar to previous studies, our results showed that males were at greater risk for developing tonsilloliths [11,21,23,24,26]. Despite discrepancies, we found an increasing trend of tonsilloliths with age, in line with several other studies [9,10,24].

Sialolith

Radiographic diagnosis of sialolith is challenging because conditions such as osteosclerosis, enostosis, osteoma, phleboliths, and lymph node calcification depicted on PRs share the same radiographic features as sialolith. Bilateral involvement of sialolith is rare and usually suggests the presence of systemic disorders [6]. Salivary calculi have been reported most commonly in patients between 30 and 60 years of age, with a higher rate of incidence in males [13,14]. We found no significant relationship between the presence of sialolith, age, and gender of the patients, although 50% of sialolith cases were found in middle-aged patients. Furthermore, sialoliths were observed to be mostly unilateral.

Calcifications in joint-related diseases

To our knowledge, none of the studies examining STCs using DPR so far have investigated the presence of calcified bodies in the TMJ. Previous TMJ-specific studies have reported its calcification to be generally unilateral and was commonly seen in women aged 40-60 years [14,15]. In the present study, 62.5% of all TMJ calcifications were in females although this relationship did not reach statistical significance. Our data support the findings of Almeida et al. who reported the presence of unilateral TMJ calcifications that were correlated with age but not gender [15].

Calcified lymph node

CLN may be related to numerous pathologies including chronic inflammation, tuberculosis, sarcoidosis, systemic sclerosis, fungal infections, rheumatoid arthritis, metastasis of calcified neoplasms, and radiotherapy. The incidence of CLN showed a significant correlation with age. The emergence of associated diseases with increasing age can be implicated in this relationship.

Osteoma cutis

Osteoma cutis, in which bone formation occurs in the skin, is a rare condition that occurs primarily in female patients and may peak in the second and third decades of life [18,24,26]. No statistically significant correlation with age or gender could be identified in this study, although 70.0% of the lesions were found in patients over 31 years of age and the female-male ratio was 9:1.

The current study showed a low incidence rate of rhinolith, in agreement with previous studies. Dacryolith, cysticercosis, metastatic calcifications, and heterotopic ossification in muscles were not detected at all in this study.

Undefined opacities

Some of the incidentally observed opacities on the examined radiographs could not be assigned to any of the known calcification groups, so we included them in the category of undefined, requiring follow-up study. The patients were unaware of the calcifications that were detected incidentally. None of the patients had any existing CT scans or other images that had been previously obtained from the region. Asymptomatic patients with undefined opacities were invited to follow-up appointments.

Multiple calcifications in the HNR often show features that are difficult to distinguish. Digital radiography offers the option of image enhancement by altering the contrast and density, which can help differentiate anatomical and pathological radiopacities. However, smaller calcifications, along with inherent limitations of panoramic imaging such as magnification, geometric distortion, patient positioning, overlapping of structures of similar densities, and anatomical noise may affect the detection of certain calcifications. Of note, different panoramic machines may show different findings because of non-standard geometries of the focal troughs. More realistic data may be obtained with multicenter large population studies carried out under identical conditions.

Diagnostic features of HNR calcifications are related to their anatomical location, morphology, distribution pattern, and intrinsic features. Therefore, dental practitioners should examine PRs closely for specific abnormalities. Despite the disadvantages mentioned, PR is used as the primary imaging modality in everyday dental practice and therefore efforts should be made to maximize its diagnostic value. Advanced imaging modalities, along with technological advances, can greatly expand the diagnostic and therapeutic possibilities for patients. However, these methods, which involve high doses of radiation, should be used sparingly and only in cases where traditional 2D imaging techniques are insufficient [29].

In recent years, deep learning-based artificial intelligence (AI) models have become the technique of choice for image analysis and have attracted enormous attention in the field of medical imaging. The application of AI models for the automatic detection of STCs using readily available PR images in dentistry may be feasible in the near future.

To our knowledge, this study incorporates the largest sample size among comparable studies to date. The absence of standardization of parameters such as selection criteria and methodology is likely to result in a wide range of prevalence of calcification in different studies.

Our study had several limitations. First, it was carried out at a single center, and the regional patient population may have different risk factors for STCs compared to other regions. Thus, the results may not be applicable to all health centers, and their validity may be limited. The second limitation is the lack of clinical diagnosis of the samples. The third drawback is a lack of comparison using different imaging methods for specific diagnoses; however, there are ethical imperatives in the justification of exposure of asymptomatic patients to electromagnetic radiation.

## Conclusions

This study showed that STCs in the HNR are relatively common and a certain percentage of the population show these calcifications on routine panoramic radiographs. This investigation identified different radiographic features of head and neck STCs, including type, bias (unilateral or bilateral), number (single or multiple), and the presence of different calcifications in the same individual. In this context, calcifications were frequently associated with age, while female patients were more likely to have calcification of certain structures. Head and neck calcifications can be followed up in an outpatient setting. Some calcifications may be harmless and may not be a cause for any concern, while others may be indicative of underlying medical conditions. Understanding the clinical significance of these calcifications and being aware of the panoramic image features can have a major impact on the quality of life of the affected patient. In striving for optimal dental treatment, this study highlights the importance of reviewing panoramic images in their entirety for the diagnosis of clinically significant findings that require referral and/or follow-up.
